# Effects of Ticagrelor versus Clopidogrel in Patients with Coronary Bifurcation Lesions Undergoing Percutaneous Coronary Intervention

**DOI:** 10.1155/2019/3170957

**Published:** 2019-03-20

**Authors:** Wei Zheng, Youmei Li, Jingdu Tian, Lufeng Li, Li Xie, Qi Mao, Wuyang Tong, Denglu Zhou, Lorenzo Azzalini, Xiaohui Zhao

**Affiliations:** ^1^Department of Cardiology, Xinqiao Hospital, Army Medical University, Third Military Medical University, Chongqing 400037, China; ^2^Division of Interventional Cardiology, Cardio-Thoracic-Vascular Department, San Raffaele Scientific Institute, Milan 20100, Italy

## Abstract

**Background:**

Percutaneous treatment of coronary bifurcation lesions can potentially lead to higher risk of ischemic events than the nonbifurcation ones, thus calling for further optimization of dual antiplatelet therapy (DAPT). This study aimed to compare the clinical outcomes from ticagrelor and clopidogrel in bifurcation lesions patients undergoing percutaneous coronary intervention (PCI).

**Methods:**

We performed a retrospective cohort study in patients with coronary bifurcation lesions. A total of 553 patients discharged on ticagrelor or clopidogrel combined with aspirin were recruited for 1-year follow-up. The incidences of primary endpoint (major adverse cardiovascular event [MACE]: a composite of cardiac death, myocardial infarction [MI] or stroke), secondary endpoints (the individual component of the primary endpoint or definite/probable stent thrombosis), and major bleeding (Bleeding Academic Research Consortium [BARC]≥3 bleeding events) were evaluated. To minimize the selection bias, a propensity score-matched population analysis was also conducted.

**Results:**

The risks of both primary endpoint (8.15% and 12.01% for the ticagrelor and clopidogrel groups, respectively; adjusted hazards ratio [HR]: 0.488, 95% confidence interval [CI]: 0.277-0.861, P=0.013) and MI (4.44% and 8.48% for the ticagrelor and clopidogrel groups, respectively; adjusted HR: 0.341, 95% CI: 0.162-0.719, P=0.005) were significantly reduced in the ticagrelor group as compared with those of the clopidogrel counterpart, whereas the risk of major bleeding was comparable (2.96% and 2.47% for the ticagrelor and clopidogrel groups, respectively; adjusted HR: 0.972, 95% CI: 0.321-2.941, P=0.960). Propensity score-matched analysis confirmed such findings.

**Conclusions:**

For patients with bifurcation lesions after PCI, ticagrelor treatment shows lower MACE and MI rates than the clopidogrel one, along with comparable major bleeding.

## 1. Introduction

Coronary bifurcations, accounting for approximately 20% of coronary lesions treated with percutaneous coronary intervention (PCI) [1], are associated with higher risk of thrombosis and worse clinical outcomes than nonbifurcation lesions. Although recent advances in drug-eluting stent technology and PCI strategies have improved the clinical outcomes of bifurcation PCI [2, 3], the rate of stent thrombosis and/or ischemic events remains considerably high [4].

Basically, the increased ischemic events after bifurcation PCI can be ascribed to multiple factors. Bifurcation lesions with multiple stent implantation or balloon inflation are associated with delay of arterial healing and platelet activation [5]. Furthermore, the low shear and flow velocity of coronary bifurcations predispose to the formation of thrombosis [6].

The optimal dual antiplatelet therapy (DAPT) regimen, consisting of aspirin and one P2Y12 inhibitor (clopidogrel, ticagrelor, or prasugrel), is the mandatory management to prevent thrombosis and ischemic events after stenting [7]. Potent P2Y12 inhibitors ticagrelor and prasugrel have been shown to be superior to clopidogrel in preventing ischemic events in patients with acute coronary syndrome (ACS) and are recommended by current guidelines to ACS patients undergoing PCI [8]. However, currently, whether ticagrelor is superior to clopidogrel in coronary bifurcation lesions is still unknown. To the best of our knowledge, there has been no such study that compared the clinical outcomes between these two kinds of P2Y12 inhibitors in coronary bifurcation patients.

To fill this knowledge gap, we herein report a systematic investigation on the impact of ticagrelor and clopidogrel on clinical outcomes of coronary bifurcations treated with PCI.

## 2. Materials and Methods

### 2.1. Study Overview and Data Acquisition

This single center-based retrospective cohort study recruited patients with bifurcation lesions undergoing PCI between June 2015 and February 2017 in Xinqiao Hospital, Chongqing, China. Patients with the following characteristics were included: reference diameter of main vessel (MV) ≥2.5 mm and the reference diameter of side branch (SB)≥2.0 mm, PCI with stent implantation in the main vessel (MV), age ≥18 years old, and discharged alive and prescribed DAPT with clopidogrel or ticagrelor and aspirin for the first 12 months after stent implantation. Patients switching between different P2Y12 inhibitors, treated with anticoagulant, not prescribed aspirin, or with malignancies were excluded.

All participants received daily aspirin of 100 mg after the intervention. In ticagrelor group, all patients received 100 mg aspirin daily plus 90 mg ticagrelor twice daily for 1 year. In clopidogrel group, all patients received 100mg aspirin plus 75 mg clopidogrel daily for 1 year. This study was approved by the institutional ethical committee of Xinqiao Hospital.

### 2.2. Definitions and Endpoints

The primary endpoint was the occurrence of a major adverse cardiovascular event (MACE) within 12 months, which is a composite of cardiac death, myocardial infarction (MI), or stroke. The secondary endpoints included the individual components of MACE or stent thrombosis. MI was defined as the elevation of cardiac biomarker values to a value above 99% of the upper reference limit and presence of one of the following: ischemic symptoms, electrocardiographic changes compatible with infarction, imaging evidence of new loss of viable myocardium or new regional wall motion abnormality, angiography, or autopsy identified intracoronary thrombus [9]. Stroke was defined as focal loss of neurologic function lasting at least 24 hours, regardless of whether the symptom was caused by an ischemic or hemorrhagic event [10]. Stent thrombosis was defined according the definitions of Academic Research Consortium (ARC) including definite and probable stent thrombosis [11].

Bleeding events were classified by Academic Research Consortium (BARC) criteria [12]. Total bleeding includes all types of BARC-defined bleeding. The primary bleeding endpoint was the appearance of BARC-defined major bleeding (BARC type ≥ 3 bleeding events). In the following 12 months, either phone interview or clinical visit was conducted to record any adverse events of the patients.

### 2.3. Statistical Analysis

Only patients who successfully completed the 1-year follow-up period were considered for analysis. Continuous variables were presented as mean±standard deviation and compared using Student's unpaired t-test. Categorical variables were presented as number with percentage and compared using chi-square test or Fisher's exact test. Multivariate Cox regression models were used to analyze the effects of ticagrelor and clopidogrel for the primary and secondary endpoints, as well as the bleeding endpoints. Covariates that were statistically significant on univariate analysis (P<0.05) and/or those that were clinically relevant were considered candidate variables in the multivariate models. The following variables were selected for analysis of the Cox proportional hazards age (<65 versus ≥65 years), sex, cardiovascular risk factors (smoking, hypertension, dyslipidemia, and diabetes), clinical history (peripheral artery disease, previous MI, and previous PCI), clinical presentation (ACS versus no-ACS), medication (renin-angiotensin system blocker, beta-blocker, and statin), bifurcation site (left main coronary artery[LM]/left anterior descending artery[LAD]/left circumflex artery[LCX], LAD/diagonal; LCX/marginal; distal right coronary artery[RCA]), true bifurcation, SYNTAX score stratification, and single-stent treatment. Adjusted hazard ratios (HRs) and 95% confidence intervals (CIs) were reported.

To minimize selection bias from the real word, additional propensity score-matched population analysis was performed. Patients with ticagrelor were matched 1:1 with patients with clopidogrel. The propensity score was estimated with a logistic regression model with the variables of age, sex, cardiovascular risk factors (smoking, hypertension, dyslipidemia, and diabetes), clinical history (peripheral artery disease, previous MI, and previous PCI), clinical presentation (ACS versus no-ACS), medication (renin-angiotensin system blocker, beta-blocker, and statin), bifurcation site (LM/LAD/LCX; LAD/diagonal; LCX/marginal; Distal RCA), true bifurcation, SYNTAX score, and single-stent treatment.

All statistical analyses were conducted with SPSS (version 25.0, IBM Corp., America). P<0.05 was considered statistically significant for all analyses.

## 3. Results

### 3.1. Characteristics of the Study Population

**Figure 1:**
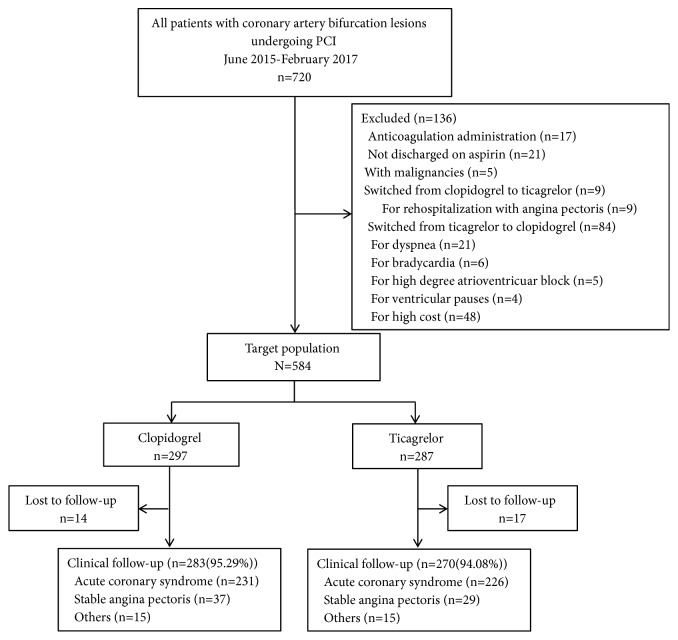
*Flow chart*. The excluded patients and subjects coincidence with the include criteria are shown. PCI: percutaneous coronary intervention.

A total of 720 bifurcation patients undergoing PCI between June 2015 and February 2017 were screened, among which 584 patients fulfilled the inclusion criteria. Of these patients, 31 (5.31%) failed the 1-year follow-up. Hence, 553 patients (283 in the clopidogrel group and 270 in the ticagrelor group) were included in the analysis. The flow chart of the study is shown in Figure 1, and the baseline characteristics of the patients are presented in Table 1. The baseline characteristics were similar between the two groups. The procedural characteristics of the study cohort are summarized in Table 2. Before the propensity score-matched analysis, the procedural characteristics were not comparable. Patients in the ticagrelor group tended to have higher prevalence of true bifurcation lesions and LM lesions and higher SYNTAX score. However, bifurcation lesion location was more likely to be the LAD/diagonal in the clopidogrel group. After propensity score matching, there were 240 patients in each group. In the matched population, no significant differences of in baseline characteristics and procedural characteristics were noted (Tables 1 and 2).

### 3.2. Primary and Secondary Endpoints for the Overall Population

**Figure 2:**
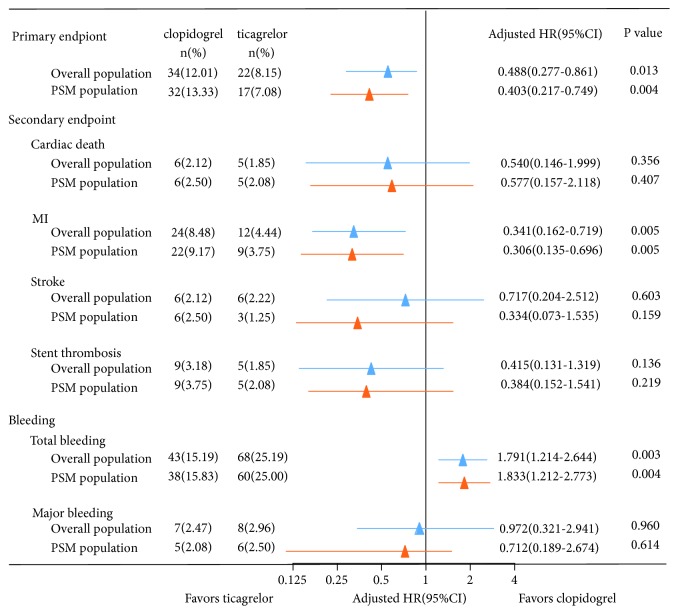
*Hazard ratios of benefit with ticagrelor versus clopidogrel for clinical outcomes in overall population and propensity score-matched population*. PSM: propensity score-matched; MI: myocardial infraction.

**Figure 3:**
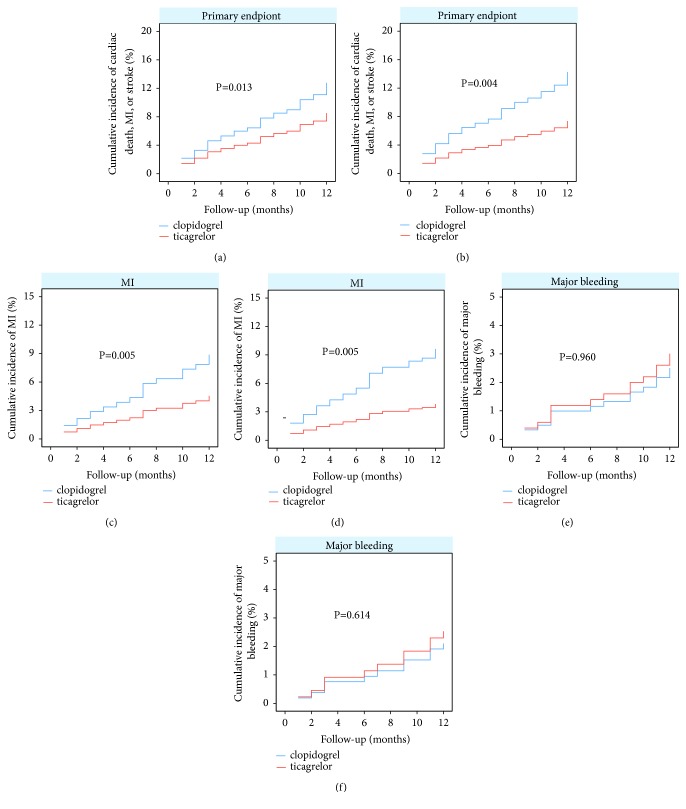
*Cumulative incidence of clinical endpionts in overall population or propensity score-matched population with bifurcation lesions undergoing PCI*. (a) and (b) Data for the primary endpoint in the overall population and the propensity score-matched population, respectively. (c) and (d) Data for the myocardial infarction (MI) in the overall population and the propensity score-matched population, respectively. (e) and (f) Data for the major bleeding in the overall population and the propensity score-matched population, respectively.

During the 1-year follow-up, the primary endpoint (cardiac death, MI, or stroke) occurred in 22 (8.15%) of 270 patients in the ticagrelor group and in 34(12.01%) of 283 patients in the clopidogrel group. MACE was less common in the ticagrelor-treated patients than the clopidogrel-treated ones (adjusted HR: 0.488, 95%CI: 0.277-0.861, P=0.013). The secondary endpoints of MI occurred in 12 (4.44%) patients in the ticagrelor group and in 24 (8.48%) patients in the clopidogrel group. The cumulative incidence of MI was lower in the ticagrelor group than that of the clopidogrel group as well (adjusted HR: 0.341, 95%CI: 0.162-0.719, P=0.005) (Table 3, Figures 2 and 3). Cardiac death occurred in 5 (1.85%) patients in the ticagrelor group and in 6 (2.12%) patients in the clopidogrel group. Stroke occurred in 6 (2.22%) patients in the ticagrelor group and in 6 (2.12%) patients in the clopidogrel group. Additionally, stent thrombosis occurred in 5 (1.85%) patients in the ticagrelor group and in 9 (3.18%) patients in the clopidogrel group. There was no notable difference in the cumulative incidence of cardiac death (adjusted HR: 0.540, 95%CI: 0.146-1.999, P=0.356), stroke (adjusted HR: 0.717, 95%CI: 0.204-2.512, P=0.603), or stent thrombosis (adjusted HR: 0.415; 95%CI: 0.131-1.319, P=0.136) (Table 3, Figure 2).

### 3.3. Bleeding for the Overall Population

The 1-year cumulative event rate of major bleeding (BARC type≥3 bleeding) was very close between the two groups (2.47 % and 2.96 % for the ticagrelor group clopidogrel groups, respectively; adjusted HR: 0.972, 95%CI: 0.321-2.941, P=0.960). The incidence of total bleeding (all BARC bleeding) was 25.19% in patients receiving ticagrelor and 15.19% in patients receiving clopidogrel. The cumulative probability of total bleeding was statistically higher in the ticagrelor group (adjusted HR: 1.791, 95%CI: 1.214-2.644, P=0.003), as shown in Table 3 and Figures 2 and 3.

### 3.4. Clinical Outcomes for the Propensity Score-Matched Population

The 1-year clinical endpoints in the 480 propensity score-matched population were similar to those in the overall population. The primary endpoint occurred in 49 patients, of them, 17 (7.08%) in the ticagrelor group and 32 (13.33%) in the clopidogrel group. The 1-year cumulative incidence of the primary endpoint was significantly lower in the ticagrelor group than in the clopidogrel group (adjusted HR: 0.403, 95%CI: 0.217-0.749, P=0.004). The cumulative incidence of MI was also significantly lower in the ticagrelor group (3.75 %) than in the clopidogrel group (9.17%) (adjusted HR: 0.306, 95%CI: 0.135-0.696, P=0.005). The risk of all BARC-defined bleeding event was higher in patients receiving ticagrelor (25.00 %) than those taking clopidogrel (15.83 %), (adjusted HR: 1.833, 95%CI: 1.212-2.773, P=0.004). Major bleeding (P=0.614) and other secondary endpoints, including cardiac death (P=0.407), stroke (P=0.159), and stent thrombosis (P=0.219) were comparable between the propensity score-matched population, as shown in Table 4 and Figures 2 and 3.

## 4. Discussion

We for the first time reported that, compared with clopidogrel, ticagrelor is associated with significantly lower risk of MACE in patients with bifurcation lesions undergoing PCI, which is probably driven by reduced MI risk.

Our results were also consistent with those of previous studies [13–16] as well as meta-analyses conducted in all-comers undergoing PCI. PLATO was a randomly controlled trial carried out with a broad population of patients with ACS, which showed reduced rates of primary composite efficacy endpoint (cardiac death, MI, or stroke), cardiac death, and MI in the ticagrelor group [13, 14]. The main finding of the SWEDEHEART registry, a real-world population based study, was that ticagrelor reduced the adjusted incidence of the composite of death, MI, or stroke relative to clopidogrel (adjusted HR, 0.85, 95%CI: 0.78–0.93)[15]. Lee et al. conducted a cohort study in Taiwan patients with acute myocardial infarction (AMI), in which a lower rate of MACE (the composite of death from any cause, AMI, or stroke) was observed at 1 year for ticagrelor-treated patients compared with clopidogrel-treated subjects [16]. In a meta-analysis of randomized controlled trials, ACS patients were found to suffer fewer MACE when treated with ticagrelor as compared with those treated with clopidogrel strategy [17]. However, it remains unclear whether patients with high risk of stent thrombosis, such as bifurcation lesions, could benefit from intense antithrombotic therapies.

Our study reveals that ticagrelor independently reduces the incidence of primary endpoint and MI for patients with bifurcation lesions undergoing PCI. The difference in primary endpoint between the two investigated groups mainly results from a significant decrease of MI with ticagrelor. Multiple mechanisms can be involved, among which platelet activation is the most probable factor for thrombosis after PCI [18]. A previous study reported that 25.7% of the PCI patients with high clopidogrel on-treatment platelet reactivity (HTPR) [19] and fewer responses to clopidogrel in patients with HTPR could be a risk factor for ischemic events, including MI [20, 21]. Therefore, the ticagrelor-mediated HTPR reduction possibly contributes to the low rate of MI in bifurcation patients undergoing stent plantation. Additionally, it was reported that ticagrelor was more efficient in improving coronary microcirculation than clopidogrel for ACS patients undergoing PCI [22]. Microcirculation dysfunction is associated with unfavorable long-term clinical outcomes after percutaneous coronary angioplasty for AMI [23].

However, the rate of stent thrombosis was slightly lower in patients treated with ticagrelor, but did not differ significantly. This phenomenon was interesting and probably resulted from the following aspects. First, stent thrombosis only took place in 5 (1.85%) of 270 patients in the ticagrelor group and in 9 (3.18%) of 283 patients in the clopidogrel group. Increasing the samples may show the potential benefit of ticagrelor in reducing stent thrombosis. Secondly, the mechanism of MI after PCI includes either disease progression or stent restenosis/thrombosis [24, 25], with stent thrombosis-generated MI only accounting for 10-18% of all MI [25–28]. Third, we found that the lesions of the ticagrelor group were more severe than those of the clopidogrel group, as evidenced by the higher SYNTAX score and higher ratio of left main bifurcation lesions. Although we applied multivariate Cox regression models and propensity score matching in the statistical analysis, the selection bias can hardly be eliminated. Hence, the residual confounding may attenuate the beneficial role of ticagrelor. Finally, MIs following stent implantation consists of non-ST-elevation myocardial infarction (NSTEMI) and ST-elevation myocardial infarction (STEMI)[25]. However, periprocedural NSTEMI accounted for most of the post-PCI MI in our cohort, whereas the most frequent mechanism of MI that presented as STEMI is stent thrombosis [26]. Therefore, the ability of ticagrelor in reducing the risk of MI probably derives from a reduced rate of NSTEMI.

Ticagrelor has shown greater platelet inhibition effect than clopidogrel, indicating better effect in preventing ischemic events of coronary artery disease. Ticagrelor plus asprin were recommended to ACS patients undergoing PCI in both drug instruction and the European Society of Cardiology (ESC) guideline [8]. However, the optional DAPT therapy for stable coronary artery disease (CAD) undergoing PCI remains unclear. ESC guideline recommends ticagrelor or prasugrel may be considered in stable coronary artery disease patients undergoing PCI with high risk of ischemic events (class IIb recommendation with level of evidence C) [8]. Existing studies about the optimal DAPT regimen in stable CAD undergoing PCI are limited. In terms of pharmacodynamic effects, ticagrelor has shown greater platelet inhibition effect than clopidogrel in stable patients [29, 30]. In terms of clinical outcomes, GLOBAL LEADERS trail had evaluated the effects of ticagrelor and clopidogrel in stable CAD patients undergoing PCI. One group treated with ticagrelor plus aspirin for 1 month, followed by ticagrelor monotherapy for 23months, the other group used aspirin plus clopidogrel for 12 months, followed by aspirin monotherapy for 12 months. Although there was no significant difference of 2-year primary endpoint between two groups [31], the duration of DAPT therapy was imbalance between the 2 groups. In our study, 37(13.07%) patients in clopidogrel group and 29 (10.07%) patients in ticagrelor group were diagnosed as stable CAD. Of them, primary endpoint took place in 4 (10.81%) patients administrated with clopidogrel and 2 (6.89%) patients administrated with ticagrelor. Ticagrelor can numerically reduce the risk of primary endpoint in stable CAD patients as compared to clopidogrel. All stable CAD patients in our study suffered coronary bifurcation lesions, indicating higher ischemic risk. So, stable CAD patients with bifurcation lesions may also obtain greater clinical benefits from ticagrelor. Large RCT trails about DAPT therapy in stable CAD are required in the future.

Bleeding is the major adverse effect of antiplatelet therapy. We found that no major bleeding (BARC type≥3) occurred frequently in the ticagrelor group, which is consistent with the finding of previous studies [13, 14, 17, 32]. In the PLATO trial, no significant difference of PLATO-defined major bleeding and coronary artery bypass graft associated major bleeding was observed between the two groups [13, 14]. In the GRAPE registry, a higher incidence of all bleeding events was seen in patients with ACS undergoing PCI who were given oral novel P2Y12 antagonist. Nevertheless, the rates of major bleeding events (BARC type≥3) were comparable for various P2Y12 inhibitors[32]. In a meta-analysis of randomized trials in patients with ACS, the frequency of major bleeding is comparable for ticagrelor and clopidogrel [17]. In contrast to the findings of present study, the SWEDEHEART registry reported a higher risk of bleeding with ticagrelor treatment. However, the proportion of long-term DAPT regimen (≥12 months) was much higher with ticagrelor than with clopidogrel (87.5% versus 61.2%), which may have contributed to the increased risk for major bleeding [15].

## 5. Limitations

The present study is a cohort study with a relatively small number of samples. Large-scale, multiple-center, randomized trials are warranted in the future. Ticagrelor is more likely to be prescribed to more severe lesions. Although multivariate analysis and propensity score-matched analysis were performed to adjust for potential confounders, hidden bias may not be excluded because of the influence of unmeasured confounders.

## 6. Conclusions

Ticagrelor enabled significantly reduced risks of MACE in patients with coronary bifurcation undergoing PCI, owing to the decreased MI risk. No significant difference in major bleeding was found between bifurcation patients treated with ticagrelor and those treated with clopidogrel. The data of our study would be useful for cardiologists because of its application in the choice of DAPT regimen in coronary bifurcation lesions after PCI.

## Figures and Tables

**Table 1:** Baseline characteristics.

Variables	Overall population			Propensity score-matched population		
	Clopidogrel (n=283)	Ticagrelor (n=270)	*P *value	Clopidogrel (n=240)	Ticagrelor (n=240)	*P *value
*Demographics*						
Age, years	64.43±11.10	63.37±10.55	0.250	64.15±11.29	64.00±10.39	0.880
Age (<65), n (%)	131(46.30)	128(47.41)	0.792	113(47.08)	108(45.00)	0.647
Women, n (%)	74(26.15)	66(24.44)	0.645	63(26.25)	61(25.42)	0.835
*Cardiovascular risk factors*						
Smoking, n (%)	136(48.06)	139(51.48)	0.421	115(47.92)	120(50.00)	0.648
Hypertension, n (%)	166(58.66)	158(58.52)	0.974	141(58.75)	137(57.08)	0.712
Dyslipidemia, n (%)	78(27.56)	80(29.63)	0.591	64(26.67)	71(29.58)	0.477
Diabetes, n (%)	74(26.15)	76(28.15)	0.597	59(24.58)	61(25.42)	0.833
*Clinical History*						
Peripheral artery disease, n (%)	56(19.79)	57(21.11)	0.700	51(21.25)	49(20.62)	0.822
Previous MI, n (%)	21(7.42)	21(7.78)	0.874	17(7.08)	18(7.50)	0.861
Previous PCI, n (%)	22(7.77)	34(12.59)	0.060	22(9.17)	20(8.33)	0.747
*Physical findings*						
Systolic Blood Pressure, mmHg	132.48±20.43	132.52±19.62	0.984	132.45±20.03	132.58±19.66	0.943
diastolic blood pressure, mmHg	77.65±12.50	77.60±12.34	0.965	78.11±12.81	77.32±12.39	0.492
Heart rates, bpm	76.26±11.81	76.47±11.45	0.830	76.65±11.93	76.25±11.54	0.709
*Clinical diagnosis*			0.698			0.550
Stable angina, n (%)	37(13.07)	29(10.74)		35(14.58)	27(11.25)	
ACS, n (%)	231(81.63)	226(83.70)		192(80.00)	200(83.33)	
Others, n (%)	15(5.30)	15(5.56)		13(5.42)	13(5.42)	
*Medication persistent to 1year*						
RAS blocker, n (%)	226(79.86)	214(79.26)	0.861	192(80.00)	189(78.75)	0.735
Beta-blocker, n (%)	198(69.96)	191(70.74)	0.842	178(74.17)	171(71.25)	0.473
Statin, n (%)	280(98.94)	269(99.63)	0.339	239(99.58)	239(99.58)	1.000

Results are expressed as mean±standard or n (%).

MI: myocardial infarction, PCI: percutaneous coronary intervention, ACS: acute coronary syndrome, and RAS: renin-angiotensin system.

**Table 2:** Procedural characteristics.

Variables	Overall population			Propensity score-matched population		
	Clopidogrel (n=283)	Ticagrelor (n=270)	*P* value	Clopidogrel (n=240)	Ticagrelor (n=240)	*P* value
*Bifurcation site*			0.003			0.063
LM/LAD/LCX, n (%)	30(10.60)	60(22.22)		30(12.50)	47(19.58)	
LAD/diagonal, n (%)	190(67.14)	162(60.00)		172(71.67)	147(61.25)	
LCX/marginal, n (%)	39(13.78)	28(10.37)		26(10.83)	27(11.25)	
Distal RCA, n (%)	24(8.48)	20(7.41)		12	19	
*Medina classification*			0.079			0.194
1.1.1, n (%)	114(40.28)	139(51.48)		106(44.17)	117(48.75)	
1.1.0, n (%)	53(18.72)	44(16.30)		32(13.33)	42(17.50)	
1.0.1, n (%)	20(7.07)	23(8.52)		18(7.50)	22(9.17)	
1.0.1, n (%)	15(5.30)	11(4.07)		10(4.17)	10(4.17)	
0.1.1, n (%)	49(17.31)	34(12.59)		47(19.58)	31(12.92)	
0.1.0, n (%)	32(11.31)	19(7.04)		27(11.25)	18(7.50)	
*True bifurcation (1.1.1, 1.0.1, 0.1.1), n (%)*	183(64.66)	196(72.59)	0.045	171(71.25)	170(70.83)	0.920
*Single stent, n (%)*	41(14.49)	45(16.67)	0.480	40(16.66)	35(14.58)	0.530
*Lesion Length*						
Main vessel, mm	15.32±5.49	14.98±5.81	0.508	15.29±5.46	14.91±5.84	0.491
Side branch, mm	9.27±3.77	9.84±4.65	0.123	9.33±3.74	9.56±4.43	0.546
*Reference vessel diameter*						
Main vessel, mm	3.00±0.38	3.07±0.42	0.055	3.03±0.38	3.07±0.42	0.290
Side branch, mm	2.37±0.37	2.43±0.44	0.056	2.39±0.38	2.43±0.44	0.243
*Stent length*						
Main vessel, mm	21.01±5.96	21.20±5.59	0.706	21.35±5.83	21.00±5.65	0.499
Side branch, mm	17.37±5.03	18.62±5.99	0.298	17.55±4.96	17.91±6.23	0.779
*Stent diameter*						
Main vessel, mm	3.19±0.39	3.25±0.41	0.122	3.23±0.39	3.25±0.42	0.681
Side branch, mm	2.85±0.34	2.92±0.43	0.381	2.84±0.35	2.93±0.48	0.377
*Syntax score*	19.41±8.44	21.01±8.62	0.028	19.92±8.61	20.17±8.00	0.740
*Syntax stratification*			0.036			0.388
1-22, n (%)	193(68.20)	161(59.63)		161(67.08)	152(63.33)	
>23, n (%)	90(31.80)	109(40.37)		79(32.92)	88(36.67)	

Results are expressed as mean± standard or n (%).

LM: left main coronary artery, LAD: left anterior descending coronary artery, LCX: left circumflex coronary artery, and RCA: right coronary artery.

**Table 3:** Clinical outcomes during 1-year follow-up in the overall population.

Overall population	Clopidogrel (n=283)	Ticagrelor (n=270)	Adjusted HR (95%CI)	*P* value
*Primary end point*				
Cardiac death, MI, or Stroke	34(12.01)	22(8.15)	0.488(0.277-0.861)	0.013
*Secondary end point*				
Cardiac death	6(2.12)	5(1.85)	0.540(0.146-1.999)	0.356
MI	24(8.48)	12(4.44)	0.341(0.162-0.719)	0.005
Stroke	6(2.12)	6(2.22)	0.717(0.204-2.512)	0.603
Stent thrombosis	9(3.18)	5(1.85)	0.415(0.131-1.319)	0.136
*Bleeding*				
All BARC type Bleeding	43(15.19)	68(25.19)	1.791(1.214-2.644)	0.003
Major bleeding	7(2.47)	8(2.96)	0.972(0.321-2.941)	0.960

Results are expressed as n (%).

MI: myocardial infarction; BARC: Bleeding Academic Research Consortium.

**Table 4:** Clinical outcomes during 1-year follow-up in the propensity score-matched population.

propensity score-matched population	Clopidogrel (n=240)	Ticagrelor (n=240)	Adjusted HR(95%CI)	*P* value
*Primary end point*				
Cardiac death, MI, or Stroke	32(13.33)	17(7.08)	0.403(0.217-0.749)	0.004
*Secondary end point*				
Cardiac death	6(2.50)	5(2.08)	0.577(0.157-2.118)	0.407
MI	22(9.17)	9(3.75)	0.306(0.135-0.696)	0.005
Stroke	6(2.50)	3(1.25)	0.334(0.073-1.535)	0.159
Stent thrombosis	9(3.75)	5(2.08)	0.384(0.152-1.541)	0.219
*Bleeding*				
All BARC type Bleeding	38(15.83)	60(25.00)	1.833(1.212-2.773)	0.004
Major bleeding	5(2.08)	6(2.50)	0.712(0.189-2.674)	0.614

Results are expressed as n (%).

For abbreviation definitions, please see the legend in Table 3.

## Data Availability

The data used to support the findings of this study are available from the corresponding author upon request.
